# Repointing and Manufacturing Instruments

**Published:** 1873-01

**Authors:** B. Bannister


### Repointing and Manufacturing Instruments.

BY B. BANNISTER.

*Read Before the Michigan State Dental Society.*

The methods here advocated, and rules recommended will be confined to manufacturing cutting instruments, and keeping them in order. Repointing or manufacturing demands of the dentist a practical knowledge of steel, in annealing, forging hardening, tempering, polishing and beveling.

What is steel?

Steel is refined iron, with carbon in proportion, of from one-half to two per cent., added to other compounds, and manufactured without carbon, with these we have nothing to do. The remarks made, the rules recommended and methods advocated, will be confined to the carbon or commercial varieties, including methods of manipulating—and principles only so far as they have proved their worth and value to the dentist. The microscope reveals that in the fracture of steel the finer the quality or more carbon steel has, the smaller are the crystals and their always taking a parellel position, according to the force given, in manufacture, from the hammer.

It also proves that under different methods of manipulating, with the hammer and temperature we may benefit, or easily destroy steel. In giving good steel a high degree of temperature, a disturbance and irregularity is occasioned in position of the crystals, or the parallellism is lost, and approaching the form or position of the poorer grades, when the crystals loose their regularity, and appear in groups or bundles, crossing each other, and of larger size.

### ANNEALING.

Steel is hardened by heating, to a certain temperature, and cooling quickly; annealed by heating to the same temperature, and cooling slowly.

Two methods are commonly used: Heat to a low red, and cool in the air, charcoal dust or ashes will answer, or heat to a low red and cool, till the color approximates the dark red, and then plunge into water.

By either method we obtain a soft condition in steel.

### FORGING.

In forging by hammering (by condensing the crystals) makes it still more fibrous, and less crystalline. The fibrous condition, gives the best cutting edge.

The more the crystalline the texture, the greater the elasticity. An application of this rule, has a practical value, in both the balde and shank, in some forms of our smaller cutting instruments, or upon any that require, the highest elastic nature with the best texture for an edge. With a proper use of the hammer, and proper temperature, we condense and elongate the crystals, improving all grades of commercial steel for cutting instruments. Heat expands, and the tendency is to displace and restore the true, crystalline form. It proves this, if we gain any benefits by condensation in our steel. We must observe care in heating to forge or harden in using the lowest practical temperature, and not destroy the fine grain, essential to the best cutting edge.

An even degree of force in blows from the hammer should be given to obtain uniformity of condensation. By continued hammering when steel is cold, the fibrous form is broken up and fractures produced.

A polished surface will take a harder and more uniform temper, with the same degree of heat, than a rough unpolished one, with the advantage of giving a better distinction of color to guide in tempering.

An even degree of heat should be given to have uniformity of hardness. In all edge tools harden thick and grind the edge afterwards. Heat, if necessary, to give proper hardness to the thicker parts. If the edge was formed before hardening the steel would burn in the thinner portion, and by loss of carbon would be converted to iron. Steel undergoing oxidation by heat, sufficient to harden, loses carbon and should be protected. By warming the surface or point, and coating with some preparation for that purpose, soap or borax will answer. A surplus of soap is to be avoided, as by taking fire, more heat will be created than is desired on the finer points. An advantage by the use of borax is, the heat is more evenly held and imparted to the steel, till the hardening bath is reached.

The temperature required to harden increases with the greater amount of carbon in the steel. In hardening we are dealing with carbon in iron. Iron cannot be given any degree of hardness till carbon is added to it. The principle if correct shows the absurdity, of the one color rule, "cherry red," to always govern in the temperature, necessary to harden the finer qualities of steel. By good primary forging, and care in heating to forge, and harden, the best results may be obtained at a lower temperature or color. By an excess of heat to harden, steel is made brittle, which is mistaken generally for hardness. By experiment such steel will be found more subject to the action of the Arkansas wheel, (a better test than the file) than if hardened at a lower degree. The success in working steel is in having a good quality, and keeping it good, in retaining the carbon and condensation by keeping down the temperature, to a practical limit in forging, in not breaking up the fibrous character, by an improper application of the hammer.

Steel hardened in water requires more heat than steel hardened in brine or any of the metallic solutions.

The greater the conductibility and density, the quicker the heat is taken from the steel. A low heat quickly absorbed, will make steel as hard as a higher heat more slowly chilled.

A practical test of this rule will convince us of the worthlessness of many of the patent tempering fluids over plain soft water. If we give steel the same degree of heat, for the different baths, we have different degrees of hardness, in proportion to the relative conducting properties of the hardening bath.

What we desire in any bath is a liquid that will rapidly take away the heat; with water, the temperature required approaches a degree, at which, unless care is observed, harm will be done the steel. In all mineral and saline solutions we may diminish the heat, but have no additional hardness, and are more liable to oxidation. In the tempering of steel after hardening, the following table indicating, by the color or oxidation of the surface, is perhaps our truest guide.

| | | | |
|---|---|---|---|
| "Very Pale | Yellow | 430 | Fahrenheit |
| | Straw | 450 | " |
| | Yellow | 470 | " |
| | Brown | 490 | " |
| Mottled | Brown | 510 | " |
| | Purple | 530 | " |
| Bright | Blue | 550 | " |
| | Blue | 560 | " |
| Dark | Blue | 600 | " |

Many of the colors we will not observe in our manipulations. Neither are they necessary, still we should give more attention than is generally done to this division. Very pale yellow and very pale straw, are our most useful colors to be left, or drawn to for all cutting edges. It is only by careful observation in good light, with the surface held upon bright steel, to compare with, that we can distinguish with accuracy the finer points in temper. Thus, is a practical worth easily detected upon the wheel or hone, between very pale straw, and yellow 40° difference in temperature, and a manifest difference in hardness.

Instruments with unequal degrees of thickness should be equally heated to harden, to avoid springing and give an even degree of strength or flexibility, on parts requiring it, when tempered.

In letting down or tempering our larger instruments having direct force, or an angle not to exceed 45°, the purple or bright blue color will be sufficient. The same class of instruments with full curve, or nearly so, will demand a still lower temper, bright blue or blue.

In the smaller straight, or instruments between 45°, and direct, bright blue will be sufficient.

For full curve or angles, approaching full, blue will be the most serviceable as also for all our smaller excavators, and hoes; dark blue has a practical value only for nerve broaches.

Different instruments require a temper suited to their form, and use. In all intermediate stages from full hardness to the lower degrees, we must be guided by form, strength, curve, and the use we subject them to.

In the polishing of steel the remarks will be confined to repointing and keeping in order of finished instruments. The method usually followed for reducing in size with files and a series of emery wheels, burnishers, etc, to produce a good surface, demands too much time, and labor. A good lathe devoted to the operating table, for repointing and keeping in order is necessary, made for the workmen to sit, and not stand to work.

The following also are requisite:

1 4 inch Very Coarse Corundum Wheel
1 3 " Turkey Stone Wheel
1 3 " Arkansas " "
1 4 " Scotch Water Stone Wheel
2 5 " Felt Wheels
1 Small Hammer
3 Polishing Sticks
1 Blowpipe, Anvil, and Lamp

I would prepare the polishing sticks by gluing well hammered sole leather to two sides. $\frac{5}{8}$inch wide $\frac{1}{2}$ inch thick—and 6 or 8 inches long for working surface, with handle, is a convenient size.

Use flour of emery, and oil upon one side. The opposite side prepare by giving a thin coat of glue, and press in No. 3 Emery, a couple of grades coarser than the flour.

The second stick of the same size, should be prepared by gluing the best French felting to two sides, using diamantine and oil, upon one, and the dry diamantine powder upon the opposite side.

The end would be of the same form, with a slight taper with a sawed slot in the end opposite to the handle, to receive emery or sand papers, cut to the form of the stick by turning back with a ferule, to slide upon, and hold the papers flat and firm to the surface.

The 5 inch felt wheels would be given the same grades of emery and preparation as the emery polishing stick.

The value of felt emery wheels is in producing a surface rapidly, with slow speed; a soft flexible working surface always conforming to any inequalities—and if not running perfect, having a continous and constant action upon the surface operated upon.

Without the wheel is elastic, it necessitates being chucked perfectly, and a speed not attainable with the majority of dental lathes.

The emery wheel or stick will rapidly remove file marks. The flour of emery wheel, removes any roughness left, by the first process, giving a surface for the burnisher or diamantine. Would burnish all soft and complete the finish with the diamantine buffer on all hardened parts, and in point of time need not exceed five minutes on any single instrument.

The emery paper stick will be serviceable on portions inaccessible with wheels or the buffers.

In repointing excavators, drills, and making nerve broaches, we may discard the use of files nearly and emery in full by the following method:

"We will repoint a hatchet excavator without annealing. I would save time and grind more smoothly. Hold my point parallel to the direction of the revolution of the corundum wheel, and when sufficient substance had been removed, would take to the Scotch water wheel, and with a few revolutions have a surface ready for the burnisher and diamantine. Anneal the point, flatten one side on the Turkey Stone Wheel; bend the point to the ground side far enough back to have when finished a round shank. Forge the blade, using the hammer evenly, and thoroughly harden, temper, and remove the oxidation by immersion in dilute sulphuric acid (to keep the surface) a few seconds, have a surface ready for the finishing process.

A continued use by this methed forms a series of grooves in the face of the Corundum and Scotch wheels, and with little attention in keeping the sizes graded, serve for forming many favorite patterns difficult to make with the file, and impossible at all times to have with the same graded uniformity and perfection.

Nerve broaches may be tempered larger and ground perfectly, ensuring a good temper, and an easy and quick method in an emergency to make what we may desire.

The object of the bevel is to form the edge. In principle its action is that of the wedge. The force is first impressed on the point of the edge or wedge to divide the substance. The character of the substance to be divided or removed whether hard or soft, demands for the best productive effect and endurence to the edge, certain angles. The bevel of all cutting instruments and drills designed for enamel or dentine, should be formed with the angles when applied to the work, as nearly parallel as possible to the surface operated upon."

How is the bevel or edge best obtained and preserved?

By discarding from the operating table the hone and using the Arkansas and Turkey wheels on the lathe. If we will examine with a glass our best efforts at honing, we may learn a good practical lesson.

The worth of many favorite instruments is in correct application of the bevel.

The hone is objectionable from the difficulty of attaining correct results, and because of the skill required and the time consumed in its use—it is almost impossible to give the same bevel at different times.

With wheels better results are obtained. Care and not skill only is necessary, and time is saved.

The obtuseness or acuteness is often to be changed in the same instrument to render its application serviceable for a particular point; this is quickly and perfectly done by the use of the wheels.

It is not economy for the dentist to manufacture, neither can we afford to be ignorant of mechanical skill, sufficient to keep our instruments in order, or to make perfectly good practical forms for our own use. Our conception of what we desire many times, and not that we have to use might be realized with a little preparation and effort to making correct pattern instruments, for the manufacturer to duplicate for us. At the chair we detect quickly the little faults and learn to prize the intrinsic worth of many instruments. It is here we can study defects, and forge in our mind, forms to be duplicated by the manufacture.

